# Single-Particle ICP-MS/MS Application for Routine Screening of Nanoparticles Present in Powder-Based Facial Cosmetics

**DOI:** 10.3390/nano13192681

**Published:** 2023-09-30

**Authors:** Deja Hebert, Jenny Nelson, Brooke N. Diehl, Phoebe Zito

**Affiliations:** 1 Department of Chemistry, University of New Orleans, New Orleans, LA 70148, USA; dghebert@uno.edu (D.H.); bdiehl@uno.edu (B.N.D.); 2 Agilent Technologies, Inc., 5301 Stevens Creek Blvd, Santa Clara, CA 95051, USA; jenny.nelson@agilent.com

**Keywords:** consumer care products, heavy metals, nanoparticles, regulations, consumer safety

## Abstract

The short- and long-term impacts of nanoparticles (NPs) in consumer products are not fully understood. Current European Union (EU) regulations enforce transparency on products containing NPs in cosmetic formulations; however, those set by the U.S. Food and Drug Administration are lacking. This study demonstrates the potential of single-particle inductively coupled plasma tandem mass spectrometry (spICP-MS/MS) as a screening method for NPs present in powder-based facial cosmetics (herein referred to as FCs). A proposed spICP-MS/MS method is presented along with recommended criteria to confirm particle presence and particle detection thresholds in seven FCs. FC products of varying colors, market values, and applications were analyzed for the presence of Bi, Cr, Mg, Mn, Pb, Sn, Ag, Al, and Zn NPs based on their ingredient lists as well as those commonly used in cosmetic formulations. The presence of NPs smaller than 100 nm was observed in all FC samples, and no correlations with their presence and market value were observed. Here, we report qualitative and semi-quantitative results for seven FC samples ranging in color, brand, and shimmer.

## 1. Introduction

Over 25 billion USD was generated in revenue by the U.S. cosmetic industry in 2021 due to increased consumer desire to improve their overall health and wellness. As a result, many cosmetic companies are now marketing their products with buzzwords such as vegan, naturally derived, eco-friendly, and cruelty-free [1] to target health-conscious consumers. This “clean beauty” marketing strategy originates from the push toward greener lifestyles and the use of environmentally safe and nontoxic products [2]. Despite their claims, clean beauty personal care products include potentially harmful chemicals, inadequate transparency on ingredient labels, and concealed substances. Cosmetic manufacturers use various ingredients to brand their products as convenient, long-lasting, and compatible with other skin applications. NPs are not the only dangerous ingredients added to FCs to enhance their physical and chemical properties. For example, cosmetics labeled as “wear-resistant” or “long-lasting” have been found to contain high concentrations of per- and polyfluoroalkyl substances (PFAs) [3]. These findings, along with others, are focused on raising awareness of the use of chemicals in personal care products and disclosing their harmful effects to consumers [4,5,6].

Common additives in various cosmetic products include minerals, vegetable powders, oils, fats, dyes and pigments, preservatives, ultraviolet filters, water, solvents, and fragrances [7]. In the U.S., the Food and Drug Administration (FDA) has lenient labeling requirements for cosmetics with regard to the disclosure of these additives and chemicals. The main ingredients required to undergo FDA approval are color additives, which are defined by the FDA as “any dye, pigment, or other substance that can impart color to a cosmetic” [8]. While the FDA does not require premarket approval for most cosmetic ingredients, they have the ability to enforce the Fair Packaging and Labeling Act (FPLA) guidelines if there are notable health concerns with a product. The FPLA states, “Informed consumers are essential to the fair and efficient functioning of a free market economy” [9]. Metalss typically in the form of metal oxides, manganese dioxide (MnO_2_), chromium oxide (CrO_3_), magnesium oxide (MgO), aluminum oxide (Al_2_O_3_), silver (Ag), bismuth oxide (Bi_2_O_3_), and tin oxide (SnO_2_) are commonly added to powder-based facial cosmetic products (herein referred to as FCs) to enhance shine, gloss, or sparkle and can act as absorbent or bulking materials [7]. To date, there are no regulations enforcing transparency on ingredient lists when it comes to the use of nanoparticles (NPs) in consumer care products. 

NPs are operationally defined as particles smaller than 100 nm in size and are added to FCs to alter their physical and chemical properties, thereby enhancing their color, longevity, and quality [10,11]. In 2009, the cosmetic industry was one of the first to incorporate NPs in consumer care products, formulating over 13% of nanotechnology-based products [12]. Notably, titanium dioxide (TiO_2_) and zinc oxide (ZnO) NPs are commonly used in sunscreen formulations as aerosols, powders, and liquids [13,14,15], potentially causing harmful effects to aquatic and human life. For example, TiO_2_ and ZnO NPs have been identified as the primary cause of the destruction of entire coral reef colonies [16]. Other studies provide evidence that NPs may cross the epithelial barrier and risk toxicity to living organisms [17,18]. This paper highlights the need for transparency regarding the use of NPs in FC products, especially for health-conscious individuals who assume their clean beauty products are nontoxic, as some ingredients may lead to negative environmental and health impacts [19,20,21].

Despite reports on their harmful effects, the use of NPs in consumer care products, especially FCs, is not common knowledge to consumers, especially those advertised with buzzwords like vegan, nontoxic, organic, or natural. Further, the FDA does not enforce the disclosure of NPs on FC ingredient lists or the use of buzzwords that create the assumption that their products are safe and healthy. The lack of transparency regarding the use of NPs is problematic and a disservice to consumers who assume clean beauty products marked as vegan or nontoxic are safe to use. In 2022, the Science Advisory Board (SAB) of the Environmental Protection Agency (EPA) released a report entitled “Review of the EPA’s Draft Fifth Contaminant Candidate List (CCL 5)” stating NPs were an emerging public health concern due to their unpredictable behavior and the overall lack of information regarding their persistence, reactivity, and short- and long-term impacts on human health and the environment [22].

All things considered, there is a large gap in the regulatory standards between the FDA and the EU on the use of nanoparticles in food and consumer care products. EU regulations enforce transparency on package labeling when NPs are added to cosmetic formulations [23]. This action prioritizes consumer awareness as to what cosmetic ingredients they are using on their bodies despite many unknown effects of NPs. Studies have shown that NPs enter aquatic environments through many sources including runoff, industrial applications and atmospheric deposition [24]. Once in the marine environment, NPs can move laterally and vertically, potentially adsorbing to organic matter [25,26,27,28], zooplankton [29,30], and other organisms [31]. 

Although numerous techniques are capable of characterizing inorganic NPs, few can disentangle NPs from other product ingredients due to their complex formulations. Other analyses to determine the size and distribution of NPs include microscopy, spectroscopy, and X-ray diffraction (XRD). However, these methods often require intense sample preparation and have low sample throughput. Previous approaches have utilized inductively coupled plasma–optical emission spectroscopy (ICP-OES) and inductively coupled plasma–mass spectrometry (ICP-MS) to detect and quantify bulk metal concentrations present in cosmetics [32,33,34]. However, studies focused on the presence, characterization, and size of NPs in FCs are lacking. Recently, the use of single-particle ICP-MS (spICP-MS) for NP detection in complex matrices has been reported [35,36,37,38]. In 2017, de la Calle et al. reported the use of spICP-MS for the screening of TiO_2_ and Au NPs in cosmetic matrices including shampoos, sunscreens, creams, and toothpastes [39]. They also emphasized the importance of consumer awareness regarding the presence of NPs in cosmetics, further validating the need for methods to identify, quantify, and characterize NPs in complex formulations. In comparison to other analytical techniques, the application of spICP-MS/MS is a relatively simple, fast, and routine analysis to observe NPs present in FC products without the need for overcomplicated sample preparation. Additionally, the utilization of tandem mass spectrometry can reduce spectral overlap and improve the limits of detection [40]. 

The purpose of this study was to utilize single-particle tandem ICP-MS (spICP-MS/MS) as a comprehensive screening tool for detecting NPs in FCs. The information gained from this study will contribute to the literature regarding the need for transparency when using additives in consumer care products and the presence of NPs in cosmetics found in local U.S. stores that are not disclosed on the packaging labels, especially those claiming to be nontoxic. The method established in this study offers a quick screening tool to analyze FCs for the presence of NPs in complex cosmetic formulations.

## 2. Materials and Methods

### 2.1. Reagents

Multielement stock solutions were purchased from Agilent and Sigma-Aldrich (Sigma-Aldrich, St. Louis, MO, USA) and were prepared in 1% (*v*/*v*) trace-metal-grade HNO_3_ (VWR, Radnor, PA, USA). Silver nanospheres (20, 50, 100, and 200 nm) suspended in sodium citrate were purchased from Nanocomposix (San Diego, CA, USA) as a reference material for single-particle size determination. Stock solutions of Ag nanospheres were diluted with nanopure water (Sartorius nanopure system, Göttingen, Germany) and prepared daily for analysis. Triton X-100 (1%) was used to prevent agglomeration and maximize the suspension of NPs present in the FC samples (Alpha Teknova, Hollister, CA, USA).

### 2.2. Cosmetics Selected for the Study 

Several cosmetic samples, in the form of eyeshadows and facial powders, were purchased from local retailers in the United States. Products were purchased to obtain a range of low-end (low cost, $) to high-end (high cost, $$$) samples. The samples contained a range of colors and shade varieties such as matte shades, glitter shades, metallic shades, and shimmer shades. Overall, seven FCs were screened for the presence of NPs (five eyeshadows and two facial powders). Sample identification and descriptions are listed in Table 1.

### 2.3. Standard and Sample Preparation

Standard solutions: Calibration standards for single-particle analysis were prepared from multielement stock solutions. Standards were prepared daily and were serially diluted by volume to a final concentration of 1 µg/L using a 1% solution of nitric acid as the diluent. The diluent was also used as the ionic blank for calibration. 

Reference materials: The silver nanoparticle reference material was diluted to concentrations between 20 and 5250 ng/L with nanopure water as the diluent. Reference materials were agitated on a shaker and sonicated after dilution to ensure particle suspension and homogeneity. Four Ag reference standards (20, 50, 100, and 200 nm) were analyzed. The final concentration of each reference standard varied by size, with larger sizes requiring higher concentrations. 

Sample Preparation: First, 0.1 g of each FC was dispersed into a 50 mL solution of 1% Triton X-100 in nanopure water. The samples were briefly shaken by hand before sonication in an ultrasonic bath for 2–3 min prior to analysis. The dilutions for each sample were made in the following ratios: 1:1, 1:3, 1:7, 1:15, 1:31, and 1:63. Each sample was shaken and sonicated in between each dilution. Samples were analyzed within 8 h of preparation to maximize particle suspension and stability.

Matrix Effect: In order to observe matrix effects, three solutions were prepared: (1) an unspiked FC sample, (2) a 1:1 dilution of an FC sample spiked with a known concentration of a 50 nm Ag NP reference material, and (3) a matrix-free reference material (RM). Each was suspended in 1% Triton X-100. Values for particle count, NPC, mass concentration, and mean size were averaged between five injections (*n* = 5). Recovery percentages were calculated based on the % recovered in the spiked sample versus the RM. 

### 2.4. Instrumentation

An Agilent 8900 ICP-MS/MS (Agilent Technologies, Santa Clara, CA, USA) equipped with nickel sampling and skimmer cones, a concentric glass nebulizer, a quartz spray chamber, and a 1.0 mm quartz torch was operated in single-particle mode. The instrument was tuned daily to optimize sensitivity. Masshunter software (Version 5.2) was used for all ICP-MS/MS analyses and data curation. The operational parameters for the analysis can be found in Table 2. Analyses were performed measuring the monitored masses (Table 2) in Time-Resolved Analysis (TRA) mode. During tandem MS, helium gas mode was utilized and both quadrupoles were set to the indicated monitored mass for on-mass measurements. The nebulization efficiency was calculated by the instrument software to obtain accurate NP sizes and elemental compositions. The nebulization efficiency is the amount of analyte that enters the plasma in relation to the amount of analyte delivered to the nebulizer. For this study, the nebulization efficiency was calculated using the Ag reference material, maintaining a value of 0.06–0.065, or 6–6.5%. The calculation was based on the particle frequency method established by Pace et al. [41].

Particle diameter was calculated by the software, which assumed particles were spherical in nature and had a specified chemical composition. The particle density and mass fraction formula of the assumed chemical composition were dependent on the particle diameter and affected the size distribution. The assumed chemical compositions in this study were chosen based on common cosmetic ingredients as well as the ingredients listed on the product packaging of the chosen samples. The assumed chemical compositions are listed in Table 3, along with their respective particle densities and mass fractions.

### 2.5. Transmission Electron Microscopy

A random sample was selected to confirm the presence of NPs in the FCs using transmission electron microscopy (TEM) to complement the spICP-MS/MS data. Sample G was dispersed in 200-proof ethanol at a concentration of 2.5 mg/mL, prepared on a Lacey/Carbon 200-mesh copper grid, and evaporated overnight. TEM images were obtained on a JEOL 2010 equipped with an EDAX genesis energy-dispersive spectroscopy (EDS) system operating at an accelerating voltage of 200 kV with an emission current of 109 μA. Figure S9 illustrates the presence of nanoparticles smaller than 100 nm.

### 2.6. Baseline and Particle Detection Threshold Determination

**Figure 1:**
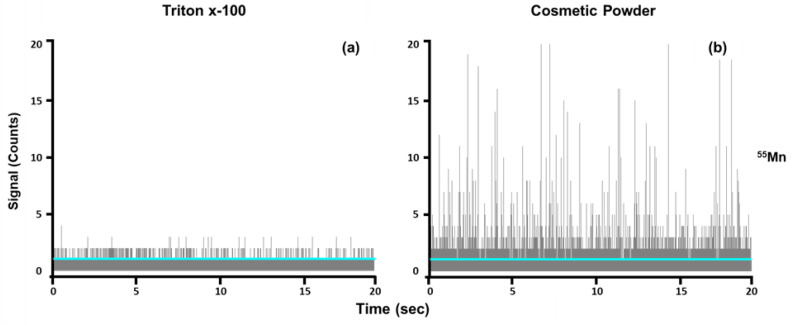
Time scans are shown for manganese. (**a**) The 1% Triton x-100 used as the blank shows no particle events, with the blue line indicating the mean intensity value. (**b**) The cosmetic powder sample diluted in 1% Triton-x 100 exhibits particle events, with the blue line indicating the baseline in the same position as the mean intensity determined for the blank.

Masshunter determined the particle baseline (Y_B_) automatically using a proprietary algorithm. The diluent (1% Triton x-100) was used as a blank. Three replicates of the blank were analyzed, and the mean intensity values for the three runs were averaged for each element. In cases where the software determined that the baseline for the samples was lower than the baseline calculated for the blank, the baseline was manually adjusted to equal the calculated mean intensity value of the blank (Figure 1). This was performed in an effort to minimize false positives so that signal intensities lower than the baseline observed in the blank were not considered as particle events.

A challenge that must be considered in single-particle analysis is the presence of particles in the blank, especially in cases where multiple elements are being screened simultaneously, thereby making it difficult to avoid the detection of particles in the blank. Therefore, a critical value for the number of particles should be determined to confirm the presence of particles in the sample at some degree higher than that in the blank. In this study, this value was calculated from the mean number of particles detected in the blank (N_B_) (Equation (1)). Particles were confirmed when the number of particles detected in the sample exceeded the value calculated using Equation (1) [43]:

N_B_ + 2.33√N_B_ (1)

Once the baseline was established and the presence of particles had been confirmed, two particle detection thresholds were calculated for each element in each sample based on Equations (2)–(5). These particle detection threshold calculations were specifically established for microsecond dwell time spICP-MS in samples with high background [44]. This technique was later applied by Vidmar et al. for the screening of nanoparticles in food matrices [35]. Thresholds I (Equation (2)) and II (Equation (3)) are considered “critical values”, where Threshold I is applied to baselines higher than five counts and Threshold II is applied to baselines lower than five counts. In calculations of Threshold I and II, only errors related to the detection of false positives are considered. Thresholds III (Equation (4)) and IV (Equation (5)) are considered “detection values” and are applied to baselines higher than five counts and lower than five counts, respectively. A more conservative approach is taken when calculating Thresholds III and IV, which account for both false positives and negatives [44].

Y_B_ + 1.64√Y_B_ (2)

Y_B_ + 2.33√Y_B_ (3)

Y_B_ + 2.71 + 3.29√Y_B_ (4)

Y_B_ + 2.71 + 4.65√Y_B_ (5)

**Figure 2:**
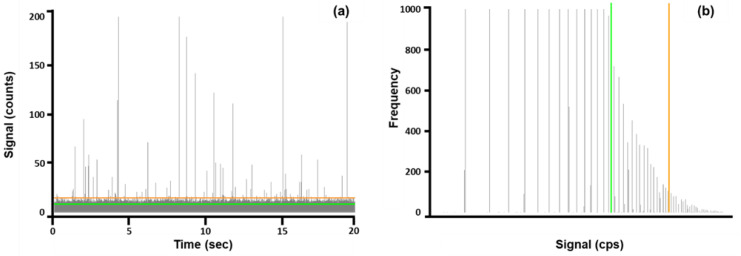
A representative time scan (**a**) and signal distribution plot (**b**) are shown for chromium present in one of the seven analyzed cosmetic samples. The green line indicates the “critical value threshold” (Threshold I), and the orange line indicates the “detection value” threshold (Threshold III). Thresholds were calculated based on Equations (2) and (4) because the determined baseline in this sample was higher than five counts.

Although the software determined a particle detection threshold, this value could be manually inputted based on user preference. In this case, all particle detection thresholds were entered manually to maintain consistent data treatment. The placement of Threshold I and Threshold III are illustrated as a representative time scan (Figure 2a) and signal distribution plot (Figure 2b) for Cr NPs detected in FCs. The signal distribution plot was translated from the time scan. 

The number of particles, limit of detection (LOD) size, and most frequent size were determined after manually entering the particle detection thresholds into the Masshunter software. Values for the calculated thresholds presented in Figure 2 are listed in Table 4.

## 3. Results and Discussion

### 3.1. spICP-MS/MS as a Screening Method for Nanoparticles in Powder-Based Facial Cosmetics

When analyzing data after an spICP-MS/MS analysis, step 1 is to confirm if particles are present in the sample. The confirmation of particle presence in spICP-MS has been referred to as “screening” in the literature [43]. Seven FC samples were screened for the presence of nine elements (Table 5), some of them disclosed on the product ingredient lists as metal oxides as well as a few commonly found in FCs. Out of the seven FC samples, only two did not contain Bi. Time scans for each element in the seven samples can be found in the Supplementary Information (Figures S1–S7), as well as time scans for each element in the blank (Figure S8).

### 3.2. spICP-MS/MS for Size Distributions

A simple screening for particles can be accomplished through a fast routine analysis, as presented in this study. However, caution must be used during data processing to ensure confidence in the determined size distributions. Microsecond dwell times and complex sample matrices are internal and external factors, respectively, that create ambiguity in the measurements. When using microsecond dwell times, multiple readings are made per particle, and those readings make up a single particle event, resulting in the calculation of a peak area [45]. Under these circumstances, the particle events are peaks rather than simple pulses. Accordingly, “pulse events” falling under the applied threshold value are eliminated from any size distribution calculations as opposed to “peak events” that are reduced in intensity based on where the threshold is applied [46]. As a result, the overall particle size distribution may be unknowingly shifted to larger sizes. Furthermore, the high complexity of the sample matrix presents a high dissolved background (Figure 2a in grey), effectively increasing the baseline, making smaller particles indistinguishable. This also causes an erroneous shift to larger particle size distributions. When this occurs, the size distribution results should be considered as “partial” unless a complementary technique is used to confirm the size distribution. 

Despite not obtaining full size distributions, size data obtained using this approach are practical for screening NPs present in FCs. Here, we provide a semi-quantitative approach using spICP-MS/MS to acquire mode particle diameters for particles present in FC products, confirming that particles exist in a sample at that size or greater. Any observations of NPs present in FCs are a testament to the applicability of this method as a screening tool, especially to satisfy criteria set by EU regulations for NPs. To minimize random error due to Poisson statistics and any bias stemming from multiple particle events, mode particle diameters are only shown for particle counts that fall between 200 and 2000. Particle counts below 200 are marked BLQ (below the limit of quantitation), and those above 2000 are marked ALQ (above the limit of quantitation). Any particle counts below the critical value calculated using Equation (1) are marked as ND (not detected). Mode diameters are reported at the more conservative of the two applied thresholds to limit bias. In addition, number particle concentrations (NPCs) calculated by the software are presented where mode diameters are reported. The calculation of the NPC is based on a formula reported elsewhere [41].

**Figure 3:**
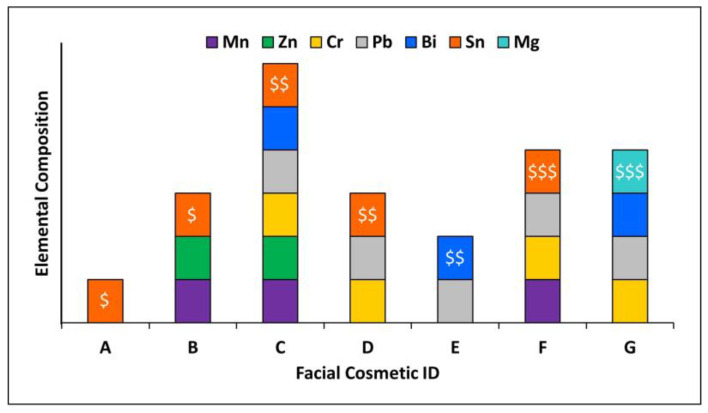
Elemental composition of particles less than 100 nm for samples A and B ($); C–E ($$); and F and G ($$$).

Semi-quantitative results for NPs detected in seven FCs using spICP-MS/MS are shown in Table 6. The concentration of aluminum particles was found to be ALQ in all samples, which was not surprising considering it is commonly used in cosmetic formulations as an “abrasive, absorbent, anticaking agent, bulking agent or opacifying agent” and has been found in products at concentrations up to 30% [47]. The amounts of Ag NPs present in FC samples A–G were BLQ, which also was not unexpected since they are added as antimicrobial agents in trace concentrations [48]. Overall, the presence and size of NPs detected in FC products varied across sample type, color, and market price value. In consideration of EU regulations, at least one element in particle form was observed below 100 nm for samples A–G (Figure 3). Sample C, a mid-cost eyeshadow ($$), and sample A, a low-cost eyeshadow ($), had the highest and lowest numbers of NPs, respectively. However, for results marked as ALQ, further sample dilutions could be performed to confirm whether the confirmed particles meet the operational definition of NPs. For results listed as BLQ, it is possible to analyze these samples at higher concentrations to increase the number of particle events. However, at such high concentrations, the background can increase, prompting the need for further sample manipulation to decrease the matrix effects, which increases the subjectivity in the measurements. None of the product packaging for the FC samples (A–G) identified any of the ingredients as being nanosized, as this is not regulated in the U.S., emphasizing that further transparency should be provided to consumers.

### 3.3. Addressing Spectral Interferences

ICP-MS continues to be a reliable technique for elemental analysis. Nonetheless, one potential challenge is the presence of spectral interferences. Spectral overlap in ICP-MS analysis can come as a result of isobaric interferences, two elements with the same isotopic mass, or polyatomic interferences, the presence of a polyatomic ion with the same mass as the measured element. Collision/reaction cell (CRC) technology has been introduced as a way to combat these spectral interferences [40,49,50,51]. Even further, when applied to a tandem mass spectrometry system, levels of sensitivity can increase significantly [52,53,54]. This combination is extremely advantageous when considering the need for elemental analysis in high-matrix samples (i.e., environmental, biological, and food) where spectral interferences are highly probable. 

Though the use of CRC technology has proven to be an effective approach to overcoming spectral interferences in normal ICP-MS analysis, its use in the single-particle mode has not been examined as extensively. Two options are available when utilizing CRC technology. The first option is the introduction of a collision gas that eliminates polyatomic interferences via kinetic energy discrimination (KED). The second option is the introduction of a reaction gas that can either react with the polyatomic interferences, which will then be filtered out by the final quadrupole, or react with the analyte ions to form an adduct ion that can be measured in “mass-shift” mode. The use of helium as a collision gas for spICP-MS measurements has been reported with demonstrated repeatability and an increase in size detection limits by 10–15% when compared with the no-gas mode [55]. The increase in size detection limits is undesirable; however, the measurement of particle types with severe spectral interferences may only be possible with the use of a collision or reaction gas. In addition, Bolea-Fernandez et al. reported on the use of different reaction gases (H_2_ (on-mass) and NH_3_ (mass-shift)) for single-particle measurements of Fe_3_O_4_ nanoparticles [56]. The results from this study revealed that the use of a heavier reaction gas like NH_3_ can induce significant peak broadening compared to lighter cell gases like H_2_ and He, leading to higher size detection limits and inaccurate size distributions. A thorough explanation of this phenomenon can be found in the referenced study [56].

In this study, there were no isobaric interferences. Common polyatomic interferences for each element are listed in Table 7 and can be found in the literature [57]. Of the possible polyatomic ions listed, those reported for Ag, Sn, Pb, and Bi seem unlikely to pose major interferences considering the sample matrix. The remaining elements (Mg, Al, Cr, Mn, and Zn) are prone to a higher number of more probable polyatomic interferences.

To examine the effects of different CRC gases, a single cosmetic sample was analyzed in three different gas modes: O_2_ mass-shift, NH_3_ mass-shift, and He on-mass. The elements analyzed were limited to Mg, Al, Cr, Mn, and Zn, as they are most likely to suffer from polyatomic interferences. To assess the effects of the three cell gases, the software-determined baselines were compared to determine their ability to decrease background presence. In addition, size detection limits (LOD_size_) were calculated for each element in the different gas modes based on the Threshold III and IV calculations described above. These values are presented in Table 8. Though Zn was analyzed, the Zn in this sample appeared to be primarily dissolved in all gas modes, as no discernible particle events were observed; therefore, values for Zn are not included. 

**Figure 4:**
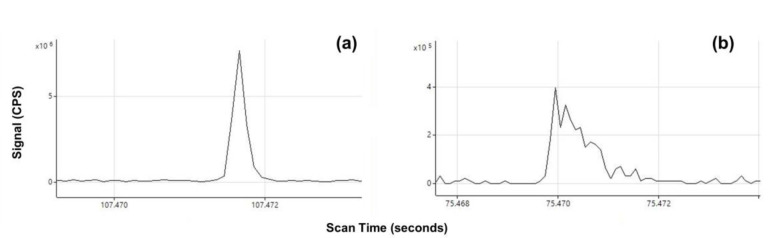
A single observed peak is shown for a magnesium particle analyzed in (**a**) He gas mode and (**b**) NH_3_ mass-shift mode. The peak observed in (**b**) exhibits increased peak width and poor peak shape.

While it is true that polyatomic interferences may contribute to an elevated background, comparing baseline values may not be the best metric for determining the optimal gas mode. The data in Table 8 show low baseline values for Mg and Al with the NH_3_ reaction gas; however, particle signals were not observed for Cr and Mn. This could be a result of the lower signal intensities typically observed in mass-shift gas modes. When comparing the LOD_size_ values, the lowest values were observed in the He gas mode despite the typically higher baseline values. The reason for this, as previously mentioned, may be peak broadening when using heavier reaction gases like NH_3_ and O_2_. An example of increased peak width and poor peak shape when using NH_3_ as a reaction gas can be seen in Figure 4b in comparison to a sharper and narrower peak observed in Figure 4a.

The use of collision and reaction cell gases has proven to be an effective measure against spectral interferences in ICP-QQQ analysis. In the case of the single-particle mode, however, the use of heavier reaction gases may increase the size detection limits, as observed in this study. Therefore, He, a lighter collision gas, was used to eliminate some spectral interferences without sacrificing peak shape, peak width, and signal intensity.

### 3.4. Importance of Dilution

**Figure 5:**
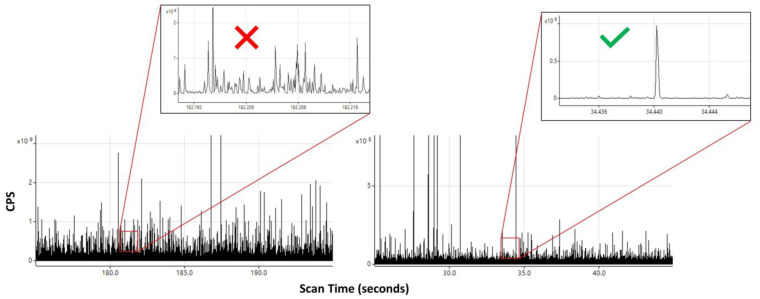
(**Left**): Time scan derived from spICP-MS/MS illustrating peak spacing issues arising from samples that are too concentrated within a 0.2 s time scan in counts per second (CPS) (inset). (**Right**): Peak spacing of a properly diluted sample within a 0.4 s time scan (inset).

It is suggested when conducting a single-particle analysis to evaluate several dilution factors, especially for samples with complex matrices. There are a number of reasons why this can be beneficial. For one, dilution of the sample also dilutes the dissolved background, effectively lowering the continuous baseline. Also, when measuring an unknown sample where the proper dilution factor is not known, the time scans of several different dilutions can be compared to determine the most accurate dilution within the acceptable range of 200–2000. When comparing time scans, an inspection of the spacing between the peak events in each dilution should be performed to ensure the most accurate results (Figure 5).

When multiple elements are measured simultaneously, the evaluation of multiple dilution factors is especially important due to the variation in concentration across different elements. Furthermore, the evaluation of multiple dilution factors during an analysis serves as a quality control. For example, size distribution is generally maintained with a concurrent decrease in particle count proportional to the dilution factor [58]. Table 9 illustrates an example of dilution effects on particle size, particle counts, and their respective dilution ratio for the Mn present in an FC sample. Particle counts over 2000 particles were observed in the first four dilutions; therefore, the likelihood of multiple particle events or particle aggregation decreased the confidence in the data. The larger particle sizes for dilutions with over 2000 particles may also be explained by the higher dissolved content increasing the baseline. The two remaining dilutions (1:31 and 1:63) were within the acceptable particle range, which was further confirmed by the proportionate decrease in particle count relative to the dilution. Perfect linearity in the particle count as a function of the dilution ratio is difficult to achieve due to the lack of homogeneity in the FCs, as observed in data collected for the third dilution (1:7). However, the general decrease in particle counts relative to the dilution ratio seems promising.

### 3.5. Matrix Matching

A common issue faced in spICP-MS research is the lack of certified reference materials available in various matrices. Unfortunately, this makes it difficult to account for the matrix effects to the full extent. One validation technique that has been proposed is the use of spiked samples [59]. In this approach, NPs of known size and composition are added to the sample matrix and the recovery of the spiked NPs is evaluated. Ideally, the recovery of the added mass should be assessed along with any variability in particle size and distribution [60]. Table 10 lists the values for particle count, NPC, mass concentration, and average mean size for a matrix-free reference material (RM), a spiked FC sample, and an unspiked FC sample. The recovery percentages for the NPC and mass concentration were 92–95% and 101–105%, respectively.

**Figure 6:**
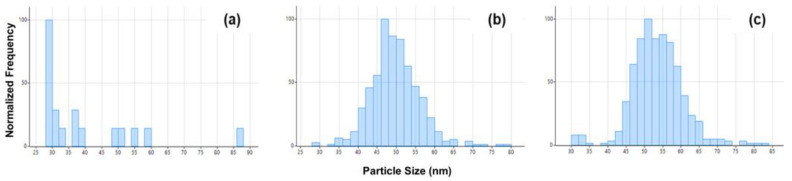
Particle size distributions for (**a**) unspiked FC sample, (**b**) matrix-free RM, and (**c**) spiked FC sample.

Figure 6 illustrates the size distribution histograms for each unspiked FC sample (Figure 6a), matrix-free RM (Figure 6b), and spiked FC (Figure 6c). Based on the calculated mean sizes and a comparison of the size distribution histograms, it appears that there was very little variability in the size distribution, as validated with the high % recovery.

Though this approach attempts to evaluate the technique’s ability to overcome matrix effects, the absence of NPs or ionic content of the same composition as the spiking material in the spiked sample may fail to mimic the true conditions of the matrix. In the case of this study, the spiked FC sample contained a minimal presence of Ag (ionic or NPs). While this may facilitate better recovery calculations, this is not a universal approach, and it cannot be applied to all NP compositions present in a sample. Ideally, a certified reference material of each NP composition would be employed for a spiked sample study; however, certified reference materials remain unavailable for use in spICP-MS.

### 3.6. Addressing the Challenges and Limitations of Screening NPs in FCs Using spICP-MS/MS

Single-particle ICP-MS/MS is a fairly recent analytical technique that is still undergoing development. That said, there are many challenges and limitations that need to be considered. A major challenge for spICP-MS/MS applications is the lack of commercially available reference materials in matrices comparable to those found in consumer products. For this reason, it is difficult to fully understand the effect of complex matrices and data derived from spICP-MS/MS analyses. Dilutions minimize matrix effects but cannot eliminate them entirely. In addition, maintaining particle stability and suspension is difficult in complex matrices where high ionic concentrations may negatively impact the results. For example, particles will settle over time during sample analysis, leading to possible inconsistencies in the particle count. Quantitative spICP-MS analysis has proven to be difficult when analyzing high-matrix samples. The elevated background may overlap with particle distributions, producing an unreliable truncated size distribution, and is dependent on the threshold detection limit (Figure 2). As mentioned, efforts to reduce elevated background include the utilization of microsecond dwell time [61], multiple sample dilutions, and tandem mass spectrometry to reduce spectral interferences. Despite utilizing these techniques, the ionic background was still often indistinguishable from the particle distribution; therefore, only qualitative and semi-quantitative results are presented.

## 4. Conclusions

An spICP-MS/MS screening method was successfully developed and applied to simultaneously identify nine different NP compositions in FC products purchased in the United States. The results from this study provide a fast, routine method using spICP-MS/MS for the screening of NPs in FC products. There were no clear trends observed between price, color, application (eyeshadow, blush, or face powder), buzzword (vegan or organic), and appearance (matte or shimmer). However, every FC sample contained NPs that were not disclosed on its ingredient list. The FDA regulates cosmetic products sold in the U.S.; however, at this time, there are no regulations addressing the use of NPs or the disclosure of NPs on packaging. Further, the use of buzzwords commonly found on cosmetic packaging may lead to the assumption that a product is nontoxic, which is misleading to health-conscious consumers given the potential for NPs to negatively impact human health and the environment.

## Figures and Tables

**Table 1:** List of sample types, cost ranges, and sample properties for each sample analyzed in this study.

Cosmetic Samples	Sample Type	Cost Range	Sample Properties
A	Eyeshadow	$	Green, Shimmer
B	Eyeshadow	$	White, Matte
C	Eyeshadow	$$	Maroon, Shimmer
D	Eyeshadow	$$	Deep Brown, Matte
E	Face Powder	$$	Pink, Shimmer
F	Eyeshadow	$$$	Green, Matte
G	Face Powder	$$$	Tan, Matte

**Table 2:** Operational parameters of the study.

Parameter	No Gas	Helium
Scan Mode	SQ	MS/MS
Gas Flow (mL/min)	0	1.0
Elements (Monitored Mass)	Al (27), Pb (208), Ag (107), Bi (209)	Mn (55), Zn (66), Cr (52), Mg (24), Sn (118)
RF Power (W)	1600	
Sampling Depth (mm)	10	
Carrier Gas (L/min)	1.20	
Dwell Time (ms)	0.1	

**Table 3:** Assumed chemical compositions for each NP type identified in this study. The mass fraction and particle density entered into the software for size calculations are listed for each NP composition. Values for NP density were obtained from the literature [42].

NP ID	Assumed NP Composition	Mass Fraction	NP Density (g/cm^3^)
Mn	MnO_2_	0.744	5.03
Zn	ZnO	0.800	5.60
Cr	Cr_2_O_3_	0.342	5.22
Mg	MgO	0.603	3.60
Al	Al_2_O_3_	0.265	3.97
Pb	PbO	0.928	9.64
Ag	Ag	1.000	10.50
Bi	Bi_2_O_3_	0.448	8.90
Sn	SnO_2_	0.788	6.85

**Table 4:** The number of particles, LOD_size_, and most frequent size are shown for the thresholds presented in Figure 2.

Threshold	Number of Particles	LOD_size_ (nm)	Most Frequent Size (nm)
1	6255	35	38
3	1225	43	46

**Table 5:** Screening results of seven cosmetic samples for nine metal particle types.

Particle Type	Number of Samples
Mn	7
Zn	7
Cr	7
Mg	7
Al	7
Pb	7
Ag	7
Bi	5
Sn	7

**Table 6:** Semi-quantitative results for particles present in seven FC samples labeled A–G.

Element ID	A		B		C		D		E		F		G	
	Mode Diameter (nm)	NPC (Particles/L)	Mode Diameter (nm)	NPC (Particles/L)	Mode Diameter (nm)	NPC (Particles/L)	Mode Diameter (nm)	NPC (Particles/L)	Mode Diameter (nm)	NPC (Particles/L)	Mode Diameter (nm)	NPC (Particles/L)	Mode Diameter (nm)	NPC (Particles/L)
Mn	BLQ		50	4.7 × 10^7^	62	7.5 × 10^7^	ALQ		50	3.1 × 10^7^	ALQ		BLQ	
Zn	BLQ		96	3.1 × 10^7^	100	9.8 × 10^7^	BLQ		BLQ		116	1.1 × 10^8^	ALQ	
Cr	BLQ		BLQ		56	4.5 × 10^7^	56	1.1 × 10^8^	50	1.8 × 10^8^	BLQ		52	8.5 × 10^7^
Mg	ALQ		ALQ		114	1.3 × 10^8^	120	2.6 × 10^8^	ALQ		ALQ		92	2.0 × 10^8^
Pb	BLQ		BLQ		30	1.1 × 10^8^	30	1.0 × 10^8^	20	7.5 × 10^7^	20	4.6 × 10^7^	20	9.2 × 10^7^
Ag	BLQ		BLQ		BLQ		BLQ		BLQ		BLQ		BLQ	
Bi	ND		ND		12	4.2 × 10^7^	ALQ		BLQ		14	2.9 × 10^7^	14	3.2 × 10^7^
Sn	66	4.6 × 10^7^	58	5.9 × 10^7^	68	5.1 × 10^7^	72	2.9 × 10^7^	66	2.3 × 10^8^	BLQ		BLQ	
Al	ALQ		ALQ		ALQ		ALQ		ALQ		ALQ		ALQ	

BLQ = below limit of quantitation; particle count < 200. ALQ = above limit of quantitation; particle count > 2000. ND = not detected.

**Table 7:** List of potential polyatomic interferences for elements analyzed in the study.

Element	Possible Interferences
^24^Mg	^12^C_2_^+^
^27^Al	^12^C^15^N^+^, ^13^C^14^N^+^, ^14^N^2^ spread, ^1^H^12^C^14^N^+^
^52^Cr	^35^Cl^16^O^1^H^+^, ^40^Ar^12^C^+^, ^36^Ar^16^O^+^, ^37^Cl^15^N^+^, ^34^S^18^O^+^, ^36^S^16^O^+^, ^38^Ar^14^N^+^, ^36^Ar^15^N^1^H^+^, ^35^Cl^17^O^+^
^55^Mn	^40^Ar^14^N^1^H^+^, ^39^K^16^O^+^, ^37^Cl^18^O^+^, ^40^Ar^15^N^+^, ^38^Ar^17^O^+^, ^36^Ar^18^O^1^H^+^ 3^8^Ar^16^O^1^H^+^, ^37^Cl^17^O^1^H^+^, ^23^Na^32^S^+^, ^36^Ar^19^F^+^
^66^Zn	^50^Ti^16^O^+^, ^34^S^16^O_2_^+^, ^33^S^16^O_2_ ^1^H^+^, ^32^S^16^O^18^O^+^, ^32^S^17^O^2+^, ^33^S^16^O^17^O^+^, ^32^S^34^S^+^, ^33^S_2_^+^
^107^Ag	^91^Zr^16^O^+^
^118^Sn	^102^Ru^16^O^+^, ^102^Pd^16^O^+^
^208^Pb	^192^Pt^16^O^+^
^209^Bi	^193^Ir^16^O^+^

**Table 8:** Comparison of baselines and size detection limits obtained for four elements suffering polyatomic interferences in three different gas modes. Scans with no particle signal are indicated by “NPS”.

Element ID	He		NH_3_			O_2_		
	Baseline (cps)	LOD_size_ (nm)	Baseline (cps)	LOD_size_ (nm)	Mass Shift	Baseline (cps)	LOD_size_ (nm)	Mass Shift
^24^Mg	111,773	125	6303	225	24 → 41	82,259	148	24 → 40
^27^Al	48,577	69	7871	92	27 → 44	97,827	96	27 → 43
^52^Cr	46,441	42	NPS	N/A	52 → 69	28,047	54	52 → 68
^55^Mn	6044	48	NPS	N/A	55→ 72	3462	64	55→ 71

**Table 9:** Quantitative results for manganese (assumed chemical composition: MnO_2_) in consecutive dilutions of an eyeshadow powder.

Dilution	Most Frequent Size (nm)	Average Size (nm)	Particle Count	Quality of Results
1:1	70	83	6837	Too many particles; high dissolved content
1:3	64	77	3866	
1:7	60	71	4263	
1:15	54	64	2977	
1:31	52	61	1570	Good particle range
1:63	52	62	828	

**Table 10:** Particle count and mean size data obtained from spiking experiment.

Sample Treatment	Particle Count	Mean Size (nm)
Matrix Free RM	513 ± 30	51 ± 1
Spiked FC Sample	478 ± 21	52 ± 1
Unspiked FC Sample	19 ± 8	42 ± 5

## Data Availability

Data are contained within the article or Supplementary Material.

## Supplementary Materials

The following supporting information can be downloaded at https://www.mdpi.com/article/10.3390/nano13192681/s1, Figure S1. Time scans are shown for the nine analyzed elements in Sample A.; Figure S2. Time scans are shown for the nine analyzed elements in Sample B; Figure S3. Time scans are shown for the nine analyzed elements in Sample C; Figure S4. Time scans are shown for the nine analyzed elements in Sample D; Figure S5. Time scans are shown for the nine analyzed elements in Sample E; Figure S6. Time scans are shown for the nine analyzed elements in Sample F; Figure S7. Time scans are shown for the nine analyzed elements in Sample G; Figure S8. Time scans are shown for the nine analyzed elements the blank 1% Triton X-100 matrix; Figure S9. Transmission electron microscopy illustrating the presence of many nanoparticles less than 100 nm for Sample G.
