# Supplementary material for: Single-Particle ICP-MS/MS Application for Routine Screening of Nanoparticles Present in Powder-Based Facial Cosmetics
Source: Nanomaterials (Basel). 2023 Sep 30;13(19):2681. doi: 10.3390/nano13192681 (PMC10574118; doi:10.3390/nano13192681)
Supplement: Supplementary file 1 [file nanomaterials-13-02681-s001.zip › nanomaterials-2585218-supplementary.pdf]

Supplementary Information

Single-Particle ICP-MS/MS Application for Routine Screening of Nanoparticles Present in Powder-Based Facial Cosmetics

Deja Hebert [1], Jenny Nelson [2], Brooke N. Diehl [1] and Phoebe Zito [1,*]

[1] Department of Chemistry, University of New Orleans, New Orleans, LA 70148, USA; dghebert@uno.edu (D.H.); bdiehl@uno.edu (B.N.D.)
[2] Agilent Technologies, Inc., 5301 Stevens Creek Blvd, Santa Clara, CA 95051, USA; jenny.nelson@agilent.com
* Correspondence: pazito@uno.edu

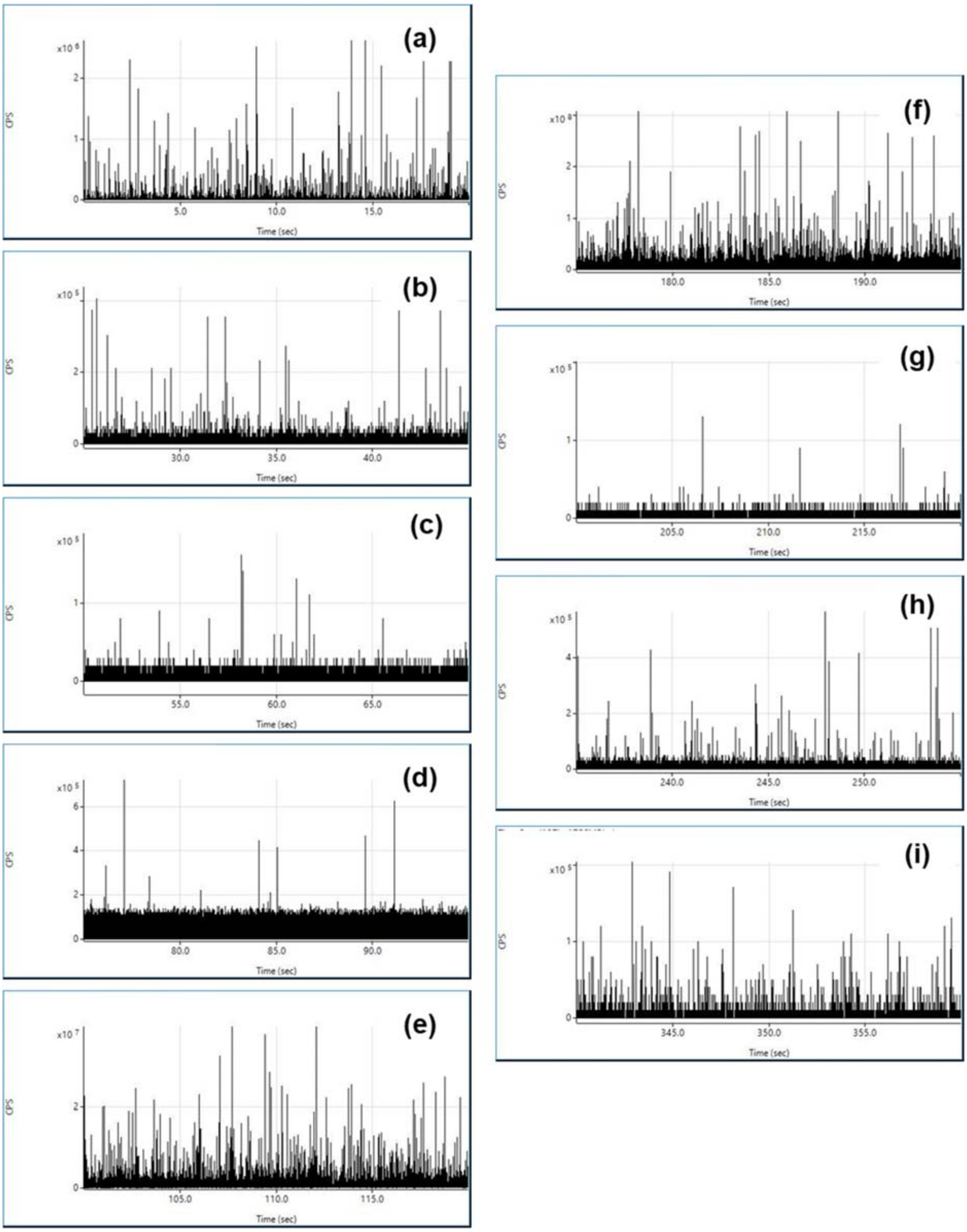

Figure S1. Time scans are shown for the nine analyzed elements in Sample A. The element represented in each time scan is as follows: (**a**) Tin (^{118}Sn) (**b**) Manganese (^{55}Mn) (**c**) Zinc (^{66}Zn) (**d**) Chromium (^{52}Cr) (**e**) Magnesium (^{24}Mg) (**f**) Aluminum (^{27}Al) (**g**) Bismuth (^{209}Bi) (**h**) Lead (^{208}Pb) (**i**) Silver (^{107}Ag).

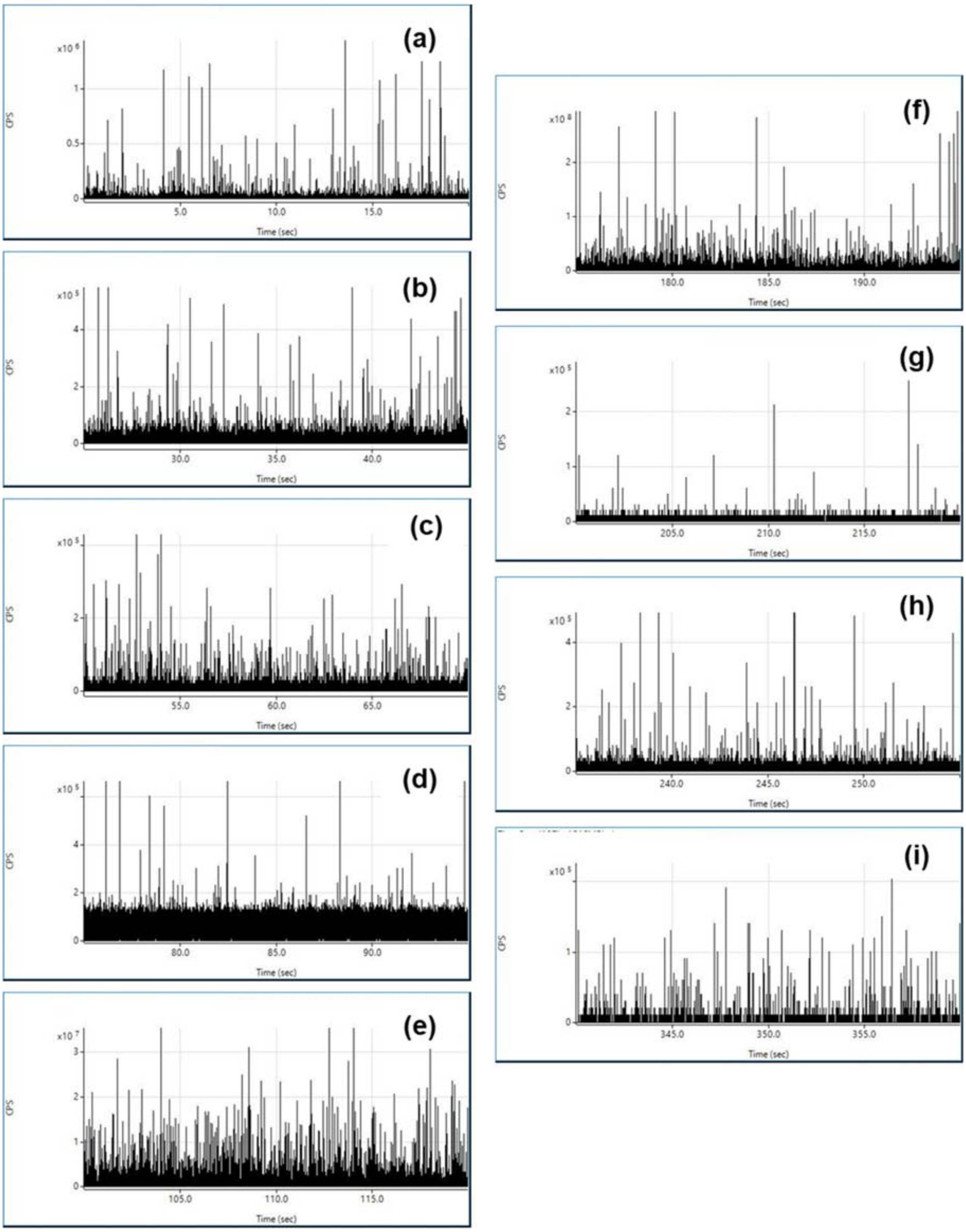

Figure S2. Time scans are shown for the nine analyzed elements in Sample B. The element represented in each time scan is as follows: (**a**) Tin (^{118}Sn) (**b**) Manganese (^{55}Mn) (**c**) Zinc (^{66}Zn) (**d**) Chromium (^{52}Cr) (**e**) Magnesium (^{24}Mg) (**f**) Aluminum (^{27}Al) (**g**) Bismuth (^{209}Bi) (**h**) Lead (^{208}Pb) (**i**) Silver (^{107}Ag).

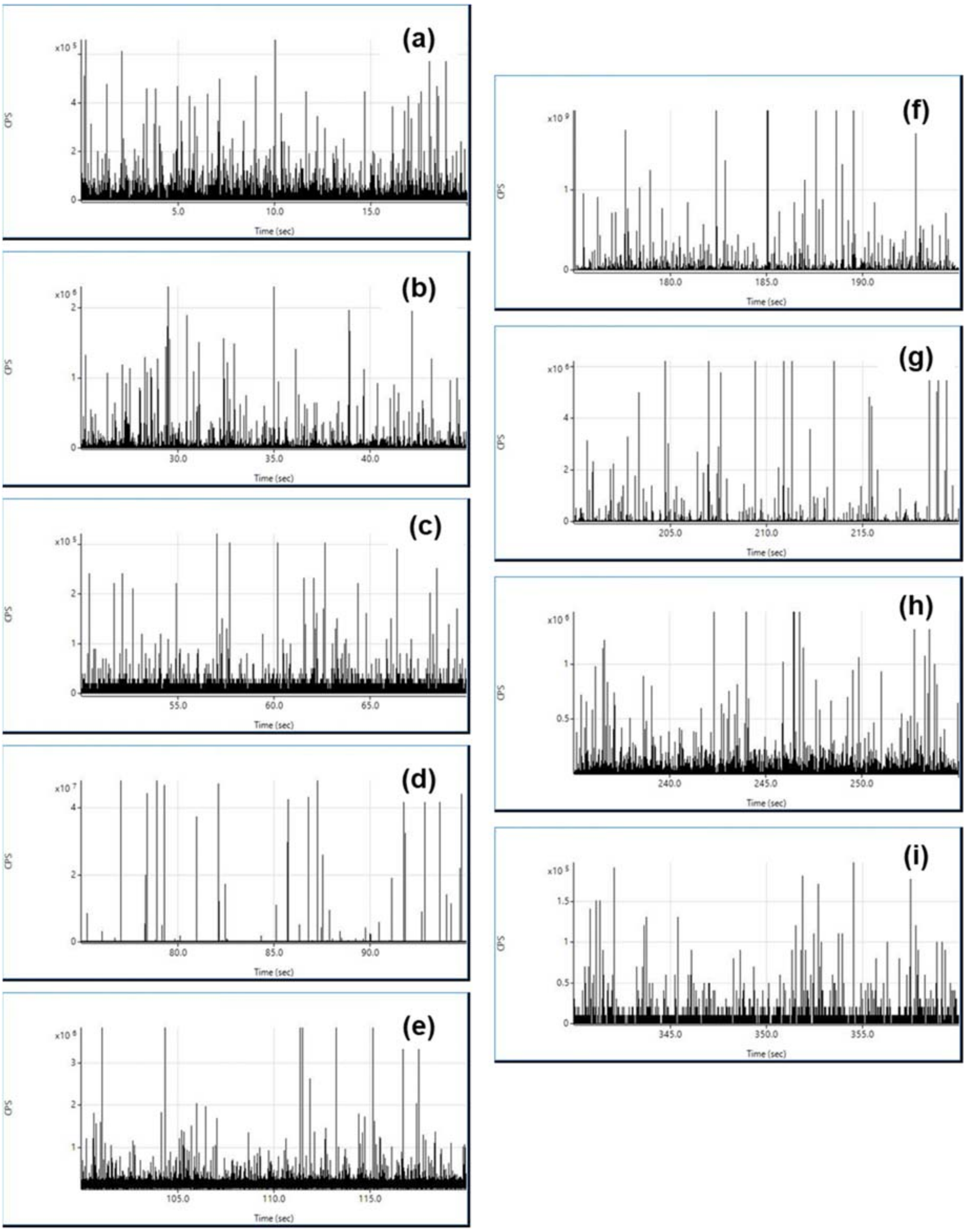

Figure S3. Time scans are shown for the nine analyzed elements in Sample C. The element represented in each time scan is as follows: (**a**) Tin (^{118}Sn) (**b**) Manganese (^{55}Mn) (**c**) Zinc (^{66}Zn) (**d**) Chromium (^{52}Cr) (**e**) Magnesium (^{24}Mg) (**f**) Aluminum (^{27}Al) (**g**) Bismuth (^{209}Bi) (**h**) Lead (^{208}Pb) (**i**) Silver (^{107}Ag).

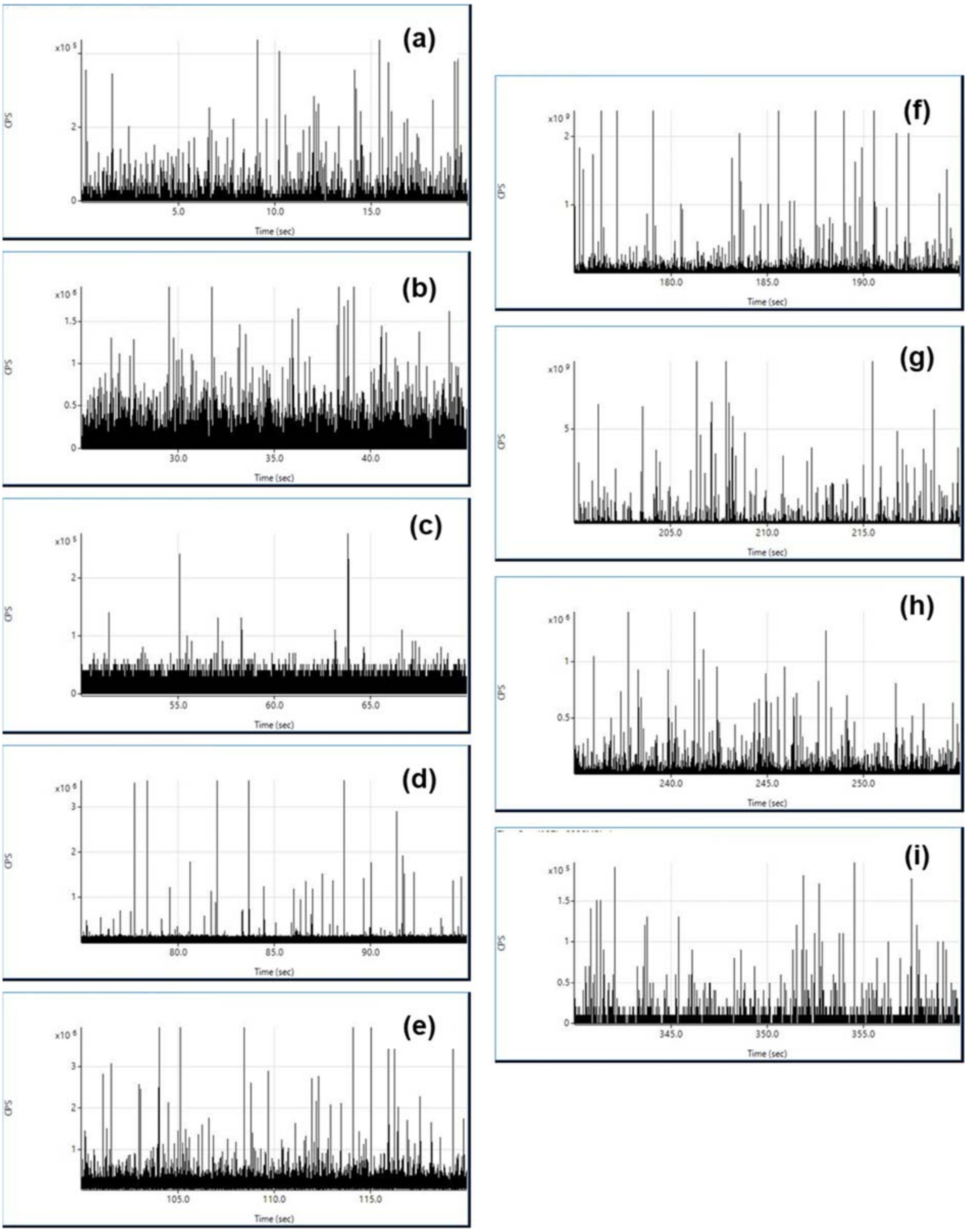

Figure S4. Time scans are shown for the nine analyzed elements in Sample D. The element represented in each time scan is as follows: (**a**) Tin (^{118}Sn) (**b**) Manganese (^{55}Mn) (**c**) Zinc (^{66}Zn) (**d**) Chromium (^{52}Cr) (**e**) Magnesium (^{24}Mg) (**f**) Aluminum (^{27}Al) (**g**) Bismuth (^{209}Bi) (**h**) Lead (^{208}Pb) (**i**) Silver (^{107}Ag).

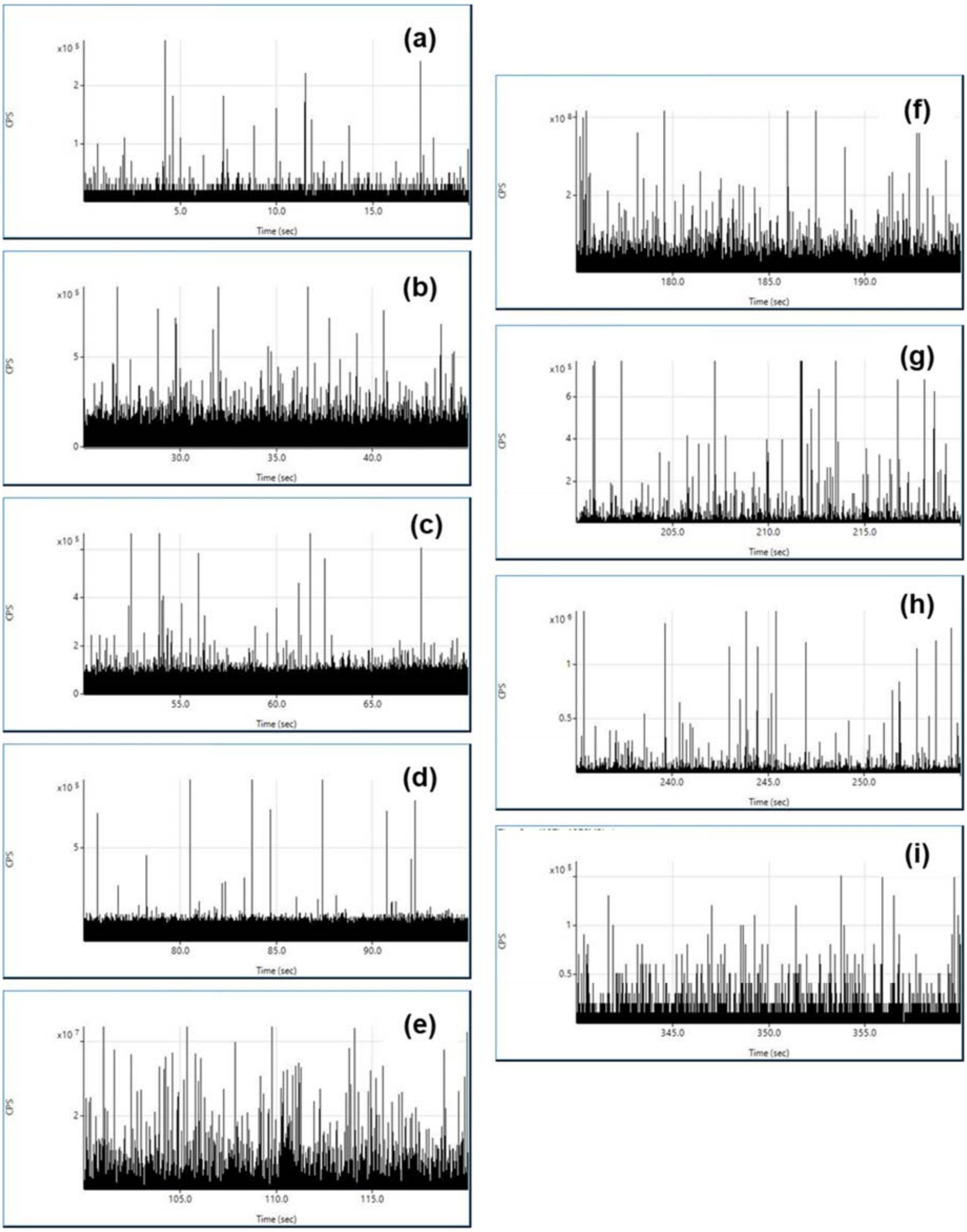

Figure S5. Time scans are shown for the nine analyzed elements in Sample E. The element represented in each time scan is as follows: (**a**) Tin (^{118}Sn) (**b**) Manganese (^{55}Mn) (**c**) Zinc (^{66}Zn) (**d**) Chromium (^{52}Cr) (**e**) Magnesium (^{24}Mg) (**f**) Aluminum (^{27}Al) (**g**) Bismuth (^{209}Bi) (**h**) Lead (^{208}Pb) (**i**) Silver (^{107}Ag).

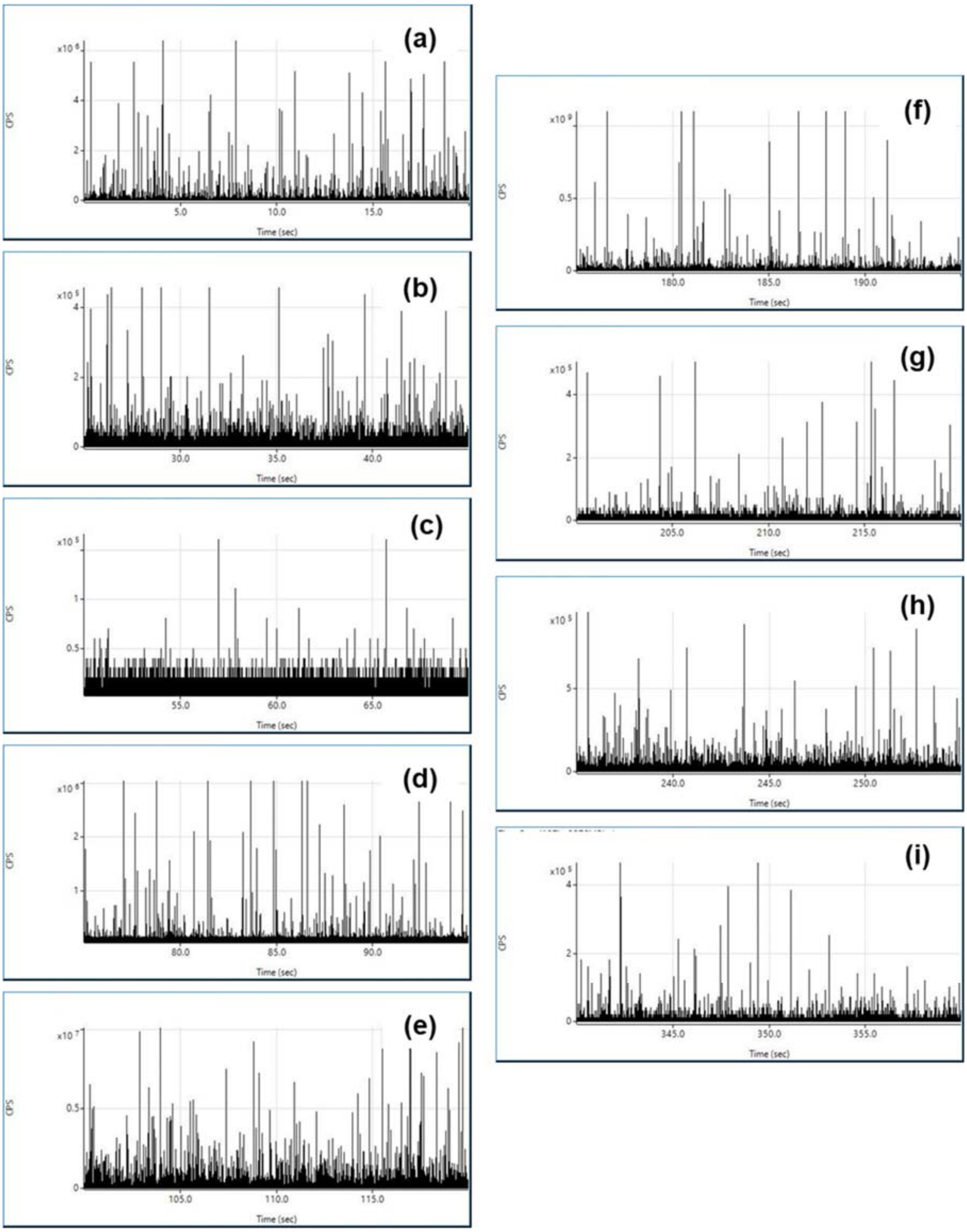

Figure S6. Time scans are shown for the nine analyzed elements in Sample F. The element represented in each time scan is as follows: (**a**) Tin (^{118}Sn) (**b**) Manganese (^{55}Mn) (**c**) Zinc (^{66}Zn) (**d**) Chromium (^{52}Cr) (**e**) Magnesium (^{24}Mg) (**f**) Aluminum (^{27}Al) (**g**) Bismuth (^{209}Bi) (**h**) Lead (^{208}Pb) (**i**) Silver (^{107}Ag).

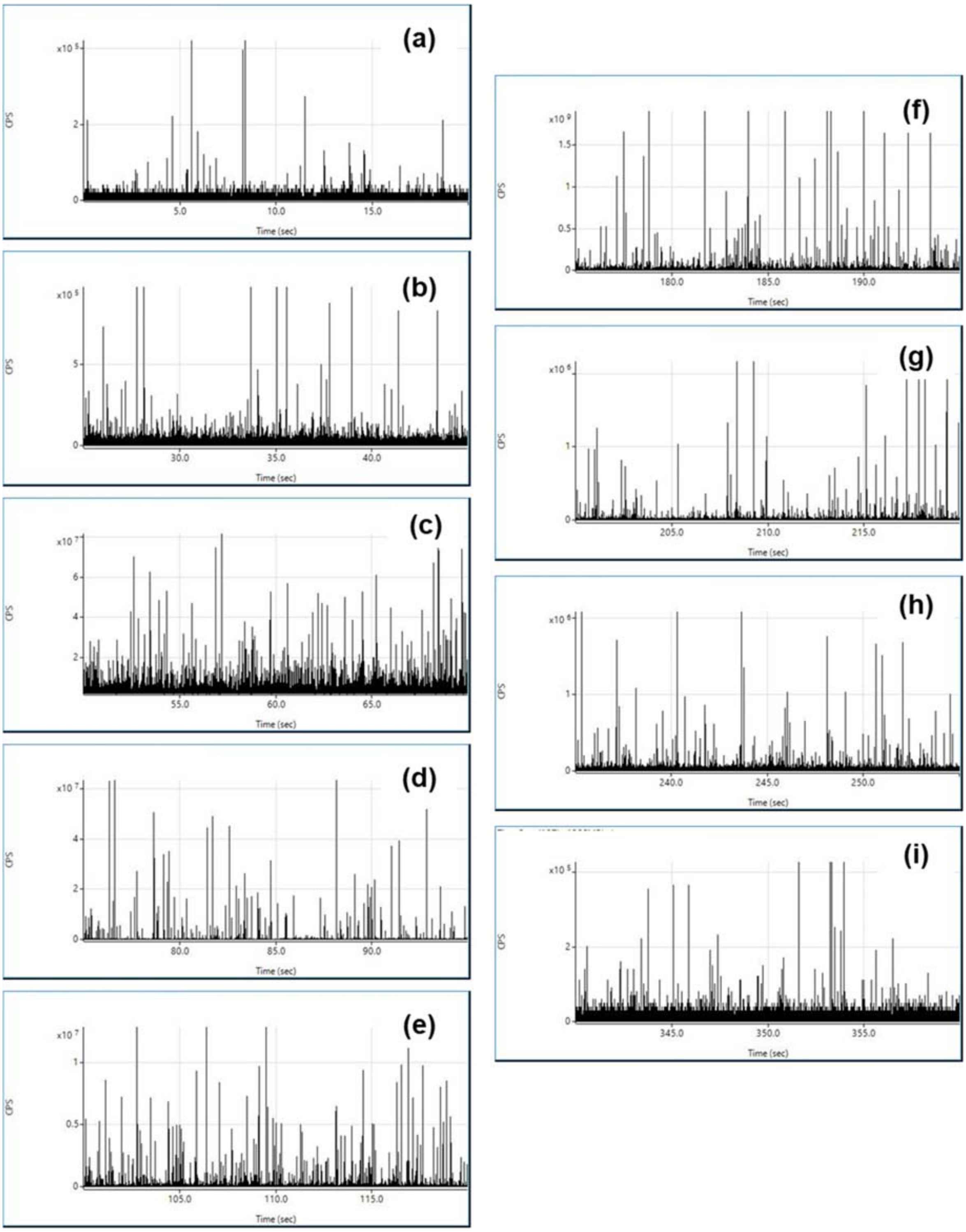

Figure S7. Time scans are shown for the nine analyzed elements in Sample G. The element represented in each time scan is as follows: (**a**) Tin (^{118}Sn) (**b**) Manganese (^{55}Mn) (**c**) Zinc (^{66}Zn) (**d**) Chromium (^{52}Cr) (**e**) Magnesium (^{24}Mg) (**f**) Aluminum (^{27}Al) (**g**) Bismuth (^{209}Bi) (**h**) Lead (^{208}Pb) (**i**) Silver (^{107}Ag).

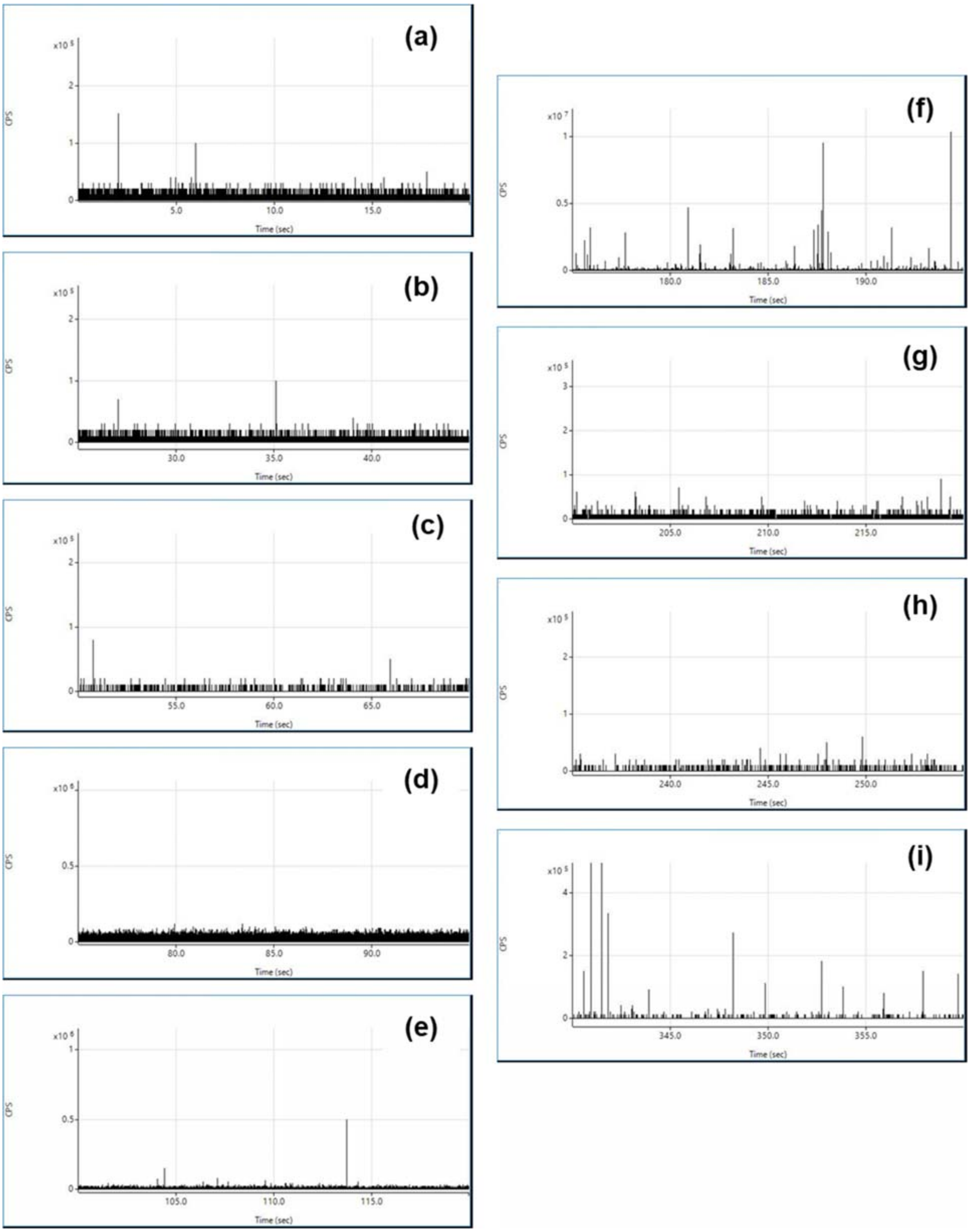

Figure S8. Time scans are shown for the nine analyzed elements the blank 1% Triton X-100 matrix. The element represented in each time scan is as follows: (**a**) Tin (^{118}Sn) (**b**) Manganese (^{55}Mn) (**c**) Zinc (^{66}Zn) (**d**) Chromium (^{52}Cr) (**e**) Magnesium (^{24}Mg) (**f**) Aluminum (^{27}Al) (**g**) Bismuth (^{209}Bi) (**h**) Lead (^{208}Pb) (**i**) Silver (^{107}Ag).

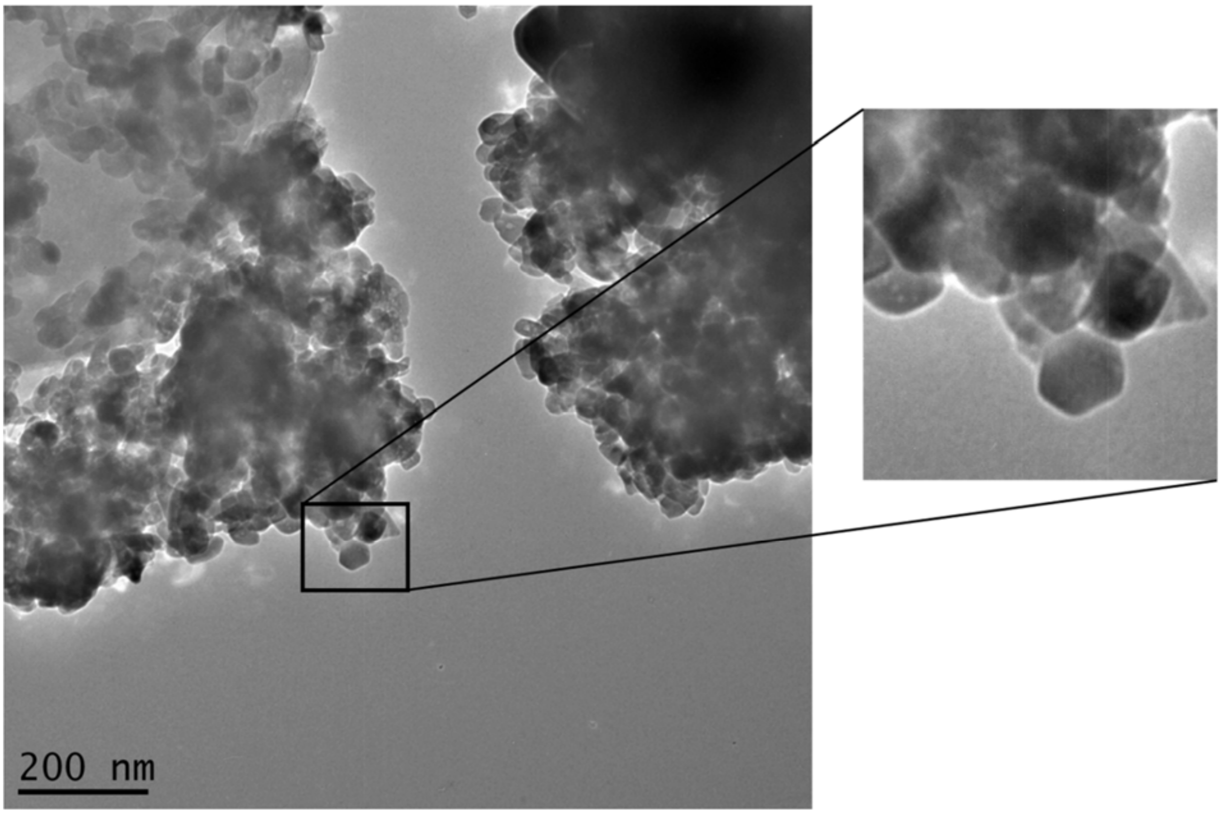

Figure S9. Transmission electron microscopy illustrating the presence of many nanoparticles less than 100 nm for Sample G.
